# Prevalence of *Chlamydia trachomatis*, *Neisseria gonorrhoeae*, *Mycoplasma genitalium* and *Ureaplasma urealyticum* infections using a novel isothermal simultaneous RNA amplification testing method in infertile males

**DOI:** 10.1186/s12941-017-0220-2

**Published:** 2017-06-24

**Authors:** Ling Qing, Qi-Xiang Song, Jian-Li Feng, Hai-Yan Li, Guiming Liu, Hai-Hong Jiang

**Affiliations:** 1 Departments of Reproductive Medicine, Urology, and Nursing, The First Affiliated Hospital of Wenzhou Medical University, #2-4P07 Nan Bai Xiang, Ouhai, Wenzhou, 325000 Zhejiang China; 2 Department of Urology, Changhai Hospital, Shanghai, 200433 China; 3 Department of Urology, The 324 Hospital of PLA, Chongqing, 400020 China; 4 Department of Surgery/Urology, MetroHealth Medical Center, Case Western Reserve University, Cleveland, OH 44109 USA

**Keywords:** Simultaneous amplification testing, *Chlamydia trachomatis*, *Neisseria gonorrhoeae*, *Mycoplasma genitalium*, *Ureaplasma urealyticum*, Male infertility, Sperm DNA fragmentation index

## Abstract

**Background:**

The purpose of this study was to evaluate the prevalence of *Chlamydia trachomatis*, *Neisseria gonorrhoeae*, *Mycoplasma genitalium* and *Ureaplasma urealyticum* infections in infertile men that consulted our outpatient departments using a novel simultaneous amplification testing (SAT) that is RNA-detection based. The possible impact of *C. trachomatis*, *N. gonorrhoeae*, *M. genitalium* and *U. urealyticum* infections on semen parameters was also noted in the present study.

**Methods:**

A total of 2607 males that were diagnosed with infertility were included in this study. *C. trachomatis*, *N. gonorrhoeae*, *M. genitalium* and *U. urealyticum* infections were detected in the urine samples using SAT method. Related data, including semen parameters and age as well as *C. trachomatis*, *N. gonorrhoeae*, *M. genitalium* and *U. urealyticum* infections were collected and analyzed.

**Results:**

A total of 51 and 1418 urine samples were found positive for *M. genitalium* RNA and *U. urealyticum* RNA, respectively, while the prevalence of *C. trachomatis* and *N. gonorrhoeae* was relatively lower. Men with positive *M. genitalium* RNA and *U. urealyticum* RNA had higher sperm DNA fragmentation index (DFI) while the comparisons of other semen parameters yielded nonsignificant results between the RNA positive and negative group. A multivariate linear regression analysis revealed that *U. urealyticum* and *M. genitalium* infections posed significant factors of DFI (adjusted R^2^ = 46.2%).

**Conclusions:**

Our study suggested a relative high prevalence of *U. urealyticum* and *M. genitalium* infection based on this novel SAT detection method. *U. urealyticum* and *M. genitalium* infection could possibly impair male fertility potential through promoting sperm DNA damage.

## Background

Male infertility is a world health problem affecting about 10–15% of couples, which accounts for half of the infertile cases [1]. The cause of male infertility has been multi-dimensional, in which the role of genitourinary tract infections has been the focus in contemporary medicine. The major genitourinary tract infections include *Chlamydia trachomatis*, *Neisseria gonorrhoeae*, mycoplasma species (*Mycoplasma genitalium* and *Mycoplasma hominis*), ureaplasma species (*Ureaplasma urealyticum* and *Ureaplasma parvum*) and *Treponema pallidum*. The exact mechanisms that genitourinary pathogens affecting male fertility potential remains unknown. The inflammatory processes triggered by genitourinary pathogens can lead to deterioration of spermatogenesis and seminal tract obstruction. The apoptosis process associated with inflammatory conditions could possibly result in the impaired semen parameters, although the relationship between the infections and semen parameters are still under debate [2].

The diagnosis of genitourinary pathogens have been based on bacterial culture, which are time consuming and fail to show adequate sensitivity. Recently, the diagnosis methods based on nucleic acid amplification methods have been widely applied in clinic, being feasible and having relative high sensitivity and specificity [3]. The first voided urine specimen has been proven be just as accurate as a urethral swab in the detection of *C. trachomatis* and *N. gonorrhoeae* [4]. Notably, a novel simultaneous amplification testing method (SAT) based on isothermal amplification of pathogens RNA has been reported providing accurate and rapid detection of several pathogens [5, 6]. To the best of our knowledge, there is no data available published regarding the prevalence of *C. trachomatis*, *N. gonorrhoeae*, *M. genitalium* and *U. urealyticum* in infertile men using this novel SAT method. Therefore, in the present study, we aimed to observe the prevalence of *C. trachomatis*, *N. gonorrhoeae*, *M. genitalium* and *U. urealyticum* in 2607 urine samples based on SAT methods of infertile men included, and to investigate the association between genitourinary infections and semen parameters. This study helps to define the diagnostic role of genitourinary infections in the assessment of male fertility potential.

## Methods

### Study population

The present multicentre study involved following medical centers: the First Affiliated Hospital of Wenzhou Medical University, Changhai Hospital, the 324 Hospital of PLA while the data was summarized and analyzed in the Case Western Reserve University and the First Affiliated Hospital of Wenzhou Medical University. From February 2016 to June 2016, we recruited males complained of infertility diagnosed with having had no pregnancies in the past of unprotected intercourse with their partners for more than 1 year that attended the outpatient department of the participated centers. All patients underwent semen analysis, semen chromatin structure assay (SCSA) analysis and *C. trachomatis*, *N. gonorrhoeae*, *M. genitalium* and *U. urealyticum* test using urine samples with SAT method. The exclusion criteria were male with reproductive system abnormalities, hormonal abnormalities, varicocele, heavy use of smoking or alcohol, exposure to physical or chemical agents with known negative reproductive effects, other causes of infertility that has been medical proven, advanced female partner age ≥38 years, detected female causes of infertility with medical evidence. Participants were also asked to confirm that they did not have any genitourinary symptoms such as pain, micturition, urethral discharge or dysuria.

### Semen analyses

Routine semen analyses were conducted by one examer according to the 4th edition of World Health Organization (WHO) laboratory manual for the examination and processing of human semen. Sperm parameters including seminal value, concentration, progressive (PR%) motility (a + b%) and normal sperm morphology were collected for further analyses. Azoospermia was defined as the absence of spermatozoa, oligospermia as the sperm concentration <20 × 10^6^/ml, asthenospermia as PR% <40%, teratospermia as normal morphology of spermatozoa <15%.

### Semen chromatin structure assay (SCSA)

Semen chromatin structure assay was performed by one examer using flow cytometry SCSA methods described previously [7]. Briefly, the acid induced sperm nuclear DNA denaturation, the semen samples were processed with acridine orange staining. Acridine orange binds to the fragmented sperm DNA that fluoresces red while the double-strand DNA fluoresces green. The SCSA parameters are calculated based on the red/(red + green) fluorescence intensity. The SCSA parameters included DFI as the percentage of the denatured sperm DNA that fluoresces red and high DNA stainability (HDS) as the percentage of sperm with abnormally high DNA statinability.

### *C. trachomatis*, *N. gonorrhoeae*, *M. genitalium* and *U. urealyticum* detection in urine samples in infertile men using SAT methods

The presence of genitourinary pathogen was carried out in urine specimens. The presence of *C. trachomatis*, *N. gonorrhoeae*, *M. genitalium* and *U. urealyticum* 16S rRNA in urine samples of infertile males, which has highly conserved sequence, were detected using SAT methods, according to the methods of the manufacture (Shanghai Rendu biotechnology Co., Ltd). Briefly, the genitourinary pathogen 16S rRNA were isolated from the sample and reverse transcribed to generate cDNA fragment. The specific 16S rRNA sense primer and anti-sense primer contains T7 promoter sequence, and is used for RNA fragment amplification. The probe sequence was labeled with 6-carboxyfluorescein (FAM) at the 5′ end and with quencher 4-[4-(dimethylamino) phenylazo] benzoic acid *N*-succinimidylester (DABCYL) at the 3′ end. Real-time PCR was performed in a real-time PCR system (Applied Biosystems Inc., Foster City, CA, USA).

### Statistical analyses

One-way Kolmogorov–Smirnov was used to test the normal distribution. Continuous variables were presented as mean ± standard deviation (SD) and compared by independent sample t test. The Chi square test or Fisher’s exact Chi square was used to for categorical variables; quantitative data non-normally distributed were presented as median (interquartile range) and compared using non-parametric test. Multivariate linear regression with likelihood ratio test was used to observe the significant predictors of DFI.

## Results

### Prevalence of *C. trachomatis*, *N. gonorrhoeae*, *M. genitalium* and *U. urealyticum* infection in infertile males

A total of 2607 urine samples of infertile males were collected and analyzed in the present study. A relative high prevalence of *U. urealyticum* was found in the detected urine samples (1418/2607, 54.5%). A total of 27 patients were positive for *C. trachomatis* (27/2607, 1.0%), 51 patients were positive for *M. genitalium* (51/2607, 2.0%), 6 patients were positive for *N. gonorrhoeae* (6/2607, 0.2%). Mix infection, defined as more than one pathogen infection, was also common in the detected samples (148/2607, 5.9%). A total of 957 samples were found negative for *C. trachomatis*, *N. gonorrhoeae, M. genitalium* or *U. urealyticum* infections (Table 1).

**Table 1:** Prevalence of CT/MG/NG/UU *C. trachomatis*, *N. gonorrhoeae*, *M. genitalium* and *U. urealyticum* in detected urine samples

	n	%
Uninfected	957	36.7
CT *C. trachomatis* only	27	1.0
MG *M. genitalium* only	51	2.0
NG *N. gonorrhoeae* only	6	0.23
UU *U. urealyticum* only	1418	54.5
Mixed infection	148	5.68

### *C. trachomatis, N. gonorrhoeae, M. genitalium* and *U. urealyticum* infection and semen parameters

The comparisons in terms of semen concentration, seminal volume, PR%, normal morphology, DFI, HDS were conducted between the pathogens positive and negative group, which were demonstrated in Table 2. The patients in *M. genitalium* positive group tended to have higher DFI% than that in *M. genitalium* negative cases (25.29 ± 15.70 versus 19.01 ± 12.80, p = 0.03). *U. urealyticum* positive subjects had about 10% higher DFI than *U. urealyticum* negative subjects (30.30 ± 16.90 versus 20.09 ± 10.56, p = 0.02). However, we failed to identify this significant differences between *C. trachomatis* positive and *C. trachomatis negative* groups, either between *N. gonorrhoeae* positive and *N. gonorrhoeae* negative groups. The mean values of seminal volume, sperm concentration, PR%, normal morphology and HDS were neither related to the detection of *C. trachomatis* RNA nor to those of *N. gonorrhoeae* or *U. urealyticum* and *M. genitalium* RNA in the detected specimens.

**Table 2:** Comparisons of semen parameters between *C. trachomatis*, *N. gonorrhoeae*, *M. genitalium* and *U. urealyticum* CT, NG, MG, UU positive and negative subjects

	Sperm concentration (×10^6^/ml)	Semen volume (ml)	PR (%)	Normal morphology (%)	DFI (%)	HDS (%)
CT *C. trachomatis*						
Positive	55.70 ± 30.80	3.18 ± 1.40	29.00 ± 19.10	9.13 ± 4.58	25.90 ± 14.45	10.15 ± 8.70
Negative	61.08 ± 40.67	3.42 ± 1.41	29.67 ± 18.78	11.45 ± 5.90	21.61 ± 10.50	12.19 ± 7.10
MG *M. genitalium*						
Positive	57.10 ± 41.40	3.51 ± 1.30	30.08 ± 18.01	12.10 ± 4.48	25.29 ± 15.70	11.65 ± 5.79
Negative	59.65 ± 41.90	3.09 ± 1.60	25.89 ± 19.01	10.10 ± 5.45	17.01 ± 12.80	15.57 ± 4.40
NG *N. gonorrhoeae*						
Positive	67.80 ± 30.90	3.78 ± 1.60	30.53 ± 17.20	15.67 ± 5.78	20.19 ± 15.67	15.00 ± 9.00
Negative	55.08 ± 30.61	3.50 ± 1.39	28.54 ± 18.34	11.45 ± 6.89	20.10 ± 15.09	13.43 ± 9.61
UU *U. urealyticum*						
Positive	50.50 ± 34.89	3.89 ± 1.21	21.90 ± 21.43	12.78 ± 6.89	30.30 ± 16.90	17.68 ± 6.05
Negative	56.90 ± 34.54	3.80 ± 1.30	21.29 ± 21.45	13.45 ± 6.89	20.09 ± 10.56	14.46 ± 5.01

The distribution of *C. trachomatis*, *N. gonorrhoeae*, *M. genitalium* and *U. urealyticum* positive cases in azoospermia versus non-azoospermia cases, oligospermia versus non-oligospermia, asthenospermia versus asthenospermia and teratospermia versus teratospermia cases were also analyzed. 2 semen specimens (2/27, 7.4%) were azoospermic in the 27 cases that were *C. trachomatis* positive while it was 11 (11/27, 40.7%), 17/27 (63.0%), 15/27 (55.6%) for oligospermia, asthenospermia and teratospermia cases, respectively. Neither *C. trachomatis* nor *N. gonorrhoeae*, *M. genitalium* or *U. urealyticum* positive was found to be related with azoospermia, oligospermia, asthenospermia or teratospermia in the current study (Table 3).

**Table 3:** The distribution of CT, NG, MG, UU positive cases in semen specimens

	Azoospermia		Oligospermia		Asthenospermia		Teratospermia	
	Yes (106)	No (2445)	Yes (1230)	No (1377)	Yes (1507)	No (1100)	Yes (1310)	No (1297)
*C. trachomatis* CT-positive (n = 27)	2 (1.88%)	25 (1.02%)	11 (0.89%)	16 (1.16%)	17 (1.13%)	10 (0.91%)	15 (1.15%)	12 (0.93%)
*M. genitalium* MG-positive (n = 51)	2 (1.88%)	49 (2.00%)	24 (1.95%)	27 (1.96%)	31 (2.06%)	20 (1.82%)	27 (2.06%)	24 (1.85%)
*N. gonorrhoeae* NG-positive (n = 51)	0 (0%)	6 (0.24%)	3 (0.24%)	3 (0.22%)	3 (0.20%)	3 (0.27%)	3 (0.23%)	3 (0.23%)
*U. urealyticum* UU-positive (n = 1418)	56 (52.8%)	1362 (55.7%)	679 (50.7%)	816 (54.1%)	816 (54.1%)	602 (54.7%)	702 (53.6%)	716 (55.2%)

### *C. trachomatis*, *N. gonorrhoeae*, *M. genitalium* and *U. urealyticum* infection and DFI elevation

DFI was the only semen parameter that correlated with pathogen infection in the current study. Hence, all parameters were introduced into multivariate linear regression analysis in the prediction of DFI. The results indicated that *U. urealyticum* and *M. genitalium* infections accounted for 46.2% of the variability in the prediction of DFI: *U. urealyticum* positive, p = 0.023; *M. genitalium* positive, p = 0.030 (Table 4).

**Table 4:** Multivariate linear regression analysis of DFI and HDS prediction

DFI%	Partial regression coefficient	SE	p	HDS%	Partial regression coefficient	SE	p
Constant	20.50	9.60	0.100		18.29	8.23	0.340
Age	0.15	2.30	0.850		0.10	0.56	0.340
UU *U. urealyticum*							
*U. urealyticum* UU-negative	Reference						
*U. urealyticum* UU-positive	8.56	5.18	0.023		−6.23	10.35	0.42
MG *M. genitalium *							
*M. genitalium * MG-negative	Reference						
*M. genitalium* MG-positive	6.26	2.45	0.030		2.20	9.35	0.59

## Discussion

Male genitourinary tract infections has always been the focus of debate in the era of male infertility. It is also estimated that approximately 15% of male infertility is related to genital tract infection [8]. *C. trachomatis*, *N. gonorrhoeae*, *M. genitalium* and *U. urealyticum* are common genitourinary tract pathogens and are widely studied in the current literature. It is also difficult to identify these infections due to their being clinically silent nature, the possibility of contamination with other organisms and the culture difficulty [9].

In our study, *C. trachomatis*, *N. gonorrhoeae*, *M. genitalium* and *U. urealyticum* infection was detected in 1.0, 2.0, 0.2 and 54.5% of infertile men, respectively. Huang et al. found that *U. urealyticum* and *M. genitalium* infections were found in 19.6 and 2.5% in infertile males, respectively [10]. *C. trachomatis* prevalence showed a wide variance, with reported rates of 0.4–42.3% in asymptomatic males in infertile couples [11]. *N. gonorrhoeae* was less evaluated in the current literature when compared to *C. trachomatis*, *M. genitalium* and *U. urealyticum*. In another study, *N. gonorrhoeae* was detected in 6.5% of infertile men, compared with 0% of fertile men [12]. These ambiguous results on the prevalence of detected pathogens can, at least partly, be the effect of differential diagnostic criteria and detection methods applied in different studies.

The consequences of genitourinary infections in the era of male fertility are still underdetermined, as well as the impact on semen parameters and sperm fertilizing capacity in the field of assisted reproductive medicine. Some studies failed to find any correlation between *C. trachomatis* infection and semen alternations [13, 14], while others reported a decrease in seminal volume, sperm concentration, motility and morphology [15–17] with *C. trachomatis* infections. Additionally, The semen quality impairments induced by *N. gonorrhoeae* and *M. genitalium* were not fully clarified in the field, with some studies reported a detrimental effect of genital pathogens on male fertility potential, while others reported altered alternation in semen parameters [2]. The heterogeneity in the male infertility diagnostic criteria and genital pathogens detection methods in different studies can partially interpret these ambiguous results. On the other hand, the effect of the presence of genital pathogens in semen on assisted reproductive technology consequences was also not fully clarified. Barbeyrac et al. found in a prospective study with 277 couples involved that the clinical pregnancy rate was comparable between the presence and absence of *C. trachomatis* infection biomarker [18]. However, in another prospective observational study, patients with *C. trachomatis* serology positive results had significant lower cumulative pregnancy rate than that in patients with *C. trachomatis* serology negative results in non-IVF treatments [19].

In our study cohort, more than half of the infertile males (54.5%) was found to have *U. urealyticum* infection. *U. urealyticum* is a natural inhabitant of the male urethra [20], while the role of *U. urealyticum* infections in male infertility pathogenesis are not fully determined. *U. urealyticum* infections has been implicated as the causative pathogen of urethritis, prostatitis and epididymitis [20]. Some researches failed to identify any correlation between *U. urealyticum* presence and semen alternations [11, 21], while others have reported a impairment on semen concentration [22], motility and morphology [11, 23]. *U. urealyticum* might have deleterious effect on sperm DNA integrity, leading to an impairment of embryo development. Sperm DNA integrity was assessed by DFI, known as sperm DNA fragmentation index, are now arising increasing attention for its diagnostic capabilities of male fertility potential and pregnancy outcome [24, 25]. *U. urealyticum* infections was found to induce sperm DNA damage and seminal reactive oxygen species and thus involved in male infertility pathogenesis in one study [26]. *U. urealyticum* was also found to cause sperm DNA denaturation both in vivo and in vitro, thus impairing embryonic development [27]. The rationality of *U. urealyticum* screening before ART cycles has also been fully acknowledged. Montagut et al. noted a significant reduction in the pregnancy rate in the *U. urealyticum* infected group [28], while there were another study reporting similar fertilization rate and pregnancy rate between the absence and presence of *U. urealyticum* in semen, although a higher abortion rate in the *U. urealyticum* positive was observed [29]. Notably, the possible influence of other detected genital pathogens infections on sperm DNA integrity had been noted in limited studies. Gallegos et al. found patients with *C. trachomatis* and *M. genitalium* infections have increased DFI and have DFI decreased from antibiotic therapy that aiming to control *C. trachomatis* and *M. genitalium* infections [30]. In our male infertility cohort, we found the routine semen parameters, including semen concentration, PR% and morphology remained unaltered regardless of *C. trachomatis, N. gonorrhoeae, M. genitalium* and *U. urealyticum* infections. However, *U. urealyticum* and *M. genitalium* infections was associated with the increase of DFI in the present study, indicating the male infertility potential impairments caused by genitourinary pathogens could possibly be mediated by a hazard impact on sperm DNA integrity.

Nucleic acid amplification tests (NAATs) has proven to provide the sensitivity, specificity and ease of specimen transport than that of any other tests available in the diagnosis of chlamydial and gonococcal infections, which was noted in the recommendations for *C. trachomatis* and *N. gonorrhoeae* detection issued by US Centers for Disease Control (CDC) and prevention [31]. Additionally, the detection progress based on RNA detection, including transcription-mediated amplification (TMA) and SAT methods has gained arising attention. TMA assay in *C. trachomatis* detection had higher sensitivity observed compared to that in DNA-based PCR detection assay [32]. This advantage of this approach is the presence of multiple copies of 16S rRNA per cell, leading to a possible higher sensitivity in comparison of PCR assays that is DNA-based that target single-copy genes. This TMA assay has proven to be the optimal methods in *M. genitalium* detection, facilitating a sensitive, specific and throughput test for MG detection [33].

Traditional methods of screening for genitourinary pathogens, like urethral swabs, are usually embarrassing and invasive, while noninvasive methods are clearly preferred by patients. Using RNA-based SAT testing method for *C. trachomatis* screening, the urine-based screening had a sensitivity and specificity 87.7 and 99.4%, respectively, which is nearly identical to those samples obtained from urethral swab (sensitivity 95.9%, specificity 99.4%) from a evidence-based medicine view [4], suggesting this urine-based noninvasive screening to be a potential alternative to invasive methods. On the other hand, the urine samples for and genitourinary pathogens detection, had been demonstrated a high concordance with semen specimens, with concordance 100% observed for *C. trachomatis*, *M. genitalium* and 85% for *U. urealyticum* detection [12]. The study of Gdoura et al. have also demonstrated a high concordance between semen and urine specimens for the detection of *C. trachomatis*, *U. urealyticum* and *M. genitalium* detection [11]. These data shed valuable light on the utility potential of urine specimens for the detection of genitourinary pathogens using SAT method, while offers a high concordance compared with semen specimens, thus facilitating the interpretation of the possible effect of these detected pathogens on semen quality and male infertility.

Several limitations should paid attention to our study. First, this is retrospective cohort study with no fertile males as “control” group included, which resulted in limited statistical power as well as provided limited information concerning the impact of *C. trachomatis*, *N. gonorrhoeae*, *M. genitalium* and *U. urealyticum* infections on male fertility potential. *U. urealyticum* and *M. genitalium* was found in 6.5 and 0.65% of the fertile males, respectively [10]. *U. urealyticum* and *M. genitalium* was found to cause sperm DFI elevation in the current study, this no “control” design makes the interpretation of the results less convincing for the prevalence of these pathogens in “control” fertile males was not detected. Second, the present study failed to compare the clinical performance in terms of prevalence, sensitivity and specificity of this novel SAT method and other existing detection method, such as bacterial culture and DNA-based assay, therefore more studies comparing this SAT and other assay are needed to uncover the advantage and disadvantage of this novel SAT method.

Despite these limitations, there are some advantages of our study that should take consideration. First, this was a cohort study with relatively large sample size (2607 cases) that evaluated *C. trachomatis*, *N. gonorrhoeae*, *M. genitalium* and *U. urealyticum* infections in infertile males and association with semen parameters. Second, to the best of our knowledge and belief, this was the first report regarding this novel SAT method using urine samples in the diagnose of genitourinary pathogens, thus providing the first hand evidence of the possible clinical utility of this SAT method. Third, the present study shed valuable light on the possibility that *M. genitalium* and *U. urealyticum* infections could cause sperm DNA damage other than impairing routine sperm parameters, thus providing the evidential proof that male genitourinary pathogens could impair male fertility potential, and this effect was possibly DFI mediated.

## Conclusions

In conclusion, using this novel SAT method, we detected a relative high prevalence of *M. genitalium* and *U. urealyticum* infections in urine samples of a infertile men cohort. Our findings indicated that *M. genitalium* and *U. urealyticum* infections could impair sperm DNA integrity, thus was likely to cause male infertility.
